# 

**Published:** 1889-02

**Authors:** 


Vol. IV. FEBRUARY, 1889. No. 8.

### DANIEL'S TEXAS MEDICAL JOURNAL

Subscription, $2.00 a Year, in Advance.—Single Copies, Twenty-five Cents.

F. E. DANIEL, M. D., Editor and Publisher, AUSTIN, TEXAS.

